# The association of multimorbidity within cardio-metabolic disease domains with dietary patterns: A cross-sectional study in 129 369 men and women from the Lifelines cohort

**DOI:** 10.1371/journal.pone.0220368

**Published:** 2019-08-08

**Authors:** Louise H. Dekker, Martin H. de Borst, Laura M. G. Meems, Rudolf A. de Boer, Stephan J. L. Bakker, Gerjan J. Navis

**Affiliations:** 1 Department of Internal Medicine, University of Groningen, University Medical Center Groningen, Groningen, the Netherlands; 2 Department of Cardiology, University of Groningen, University Medical Center Groningen, Groningen, the Netherlands; Medizinische Universitat Graz, AUSTRIA

## Abstract

**Background:**

Multimorbidity is considered a major challenge for current health care. Lifestyle interventions, as a broad and generic approach, may have the potential to improve the management of care among patients with multimorbidity. The objective of this study was to evaluate the association of multimorbidity defined within the cardiometabolic disease domains with dietary patterns, representing habitual dietary intake.

**Design:**

We studied 129 369 participants from the Lifelines Cohort study (42% male, 45±13 years (range 18–93)) in which diet was assessed using a 110-item food frequency questionnaire. A composite morbidity score was applied in multivariable ordered logistic regression to test the association with dietary patterns derived by principal components analysis, based on sex-specific dietary pattern scores.

**Results:**

Four dietary patterns were retained, accounting for 26.6% of the variation in overall diet. After control for potential confounders, men and women in the highest quintile of “meat, alcohol and potato pattern” and “snack pattern” had a higher likelihood of having higher morbidity scores than those in the lowest quintile (e.g. men: OR = 1.83(95% CI:1.71–1.97), OR = 1.18(95% CI 1.11–1.27 respectively). The opposite was observed with respect to the “bread and sweets pattern” and “vegetable, fish and fruit pattern” (e.g. women: OR = 0.88(95% CI: 0.81–0.96), OR = 0.86(95% CI 0.81–0.92 respectively). The association partially attenuated after adjusting for BMI, but the associations remained significant among men.

**Conclusions:**

Robust associations between dietary patterns and multimorbidity within the cardiometabolic domain, in particular a “meat, alcohol and potato pattern”, suggest an important opportunity of dietary interventions in multimobidity prevention. Generic prevention strategies based on population derived dietary patterns may have the potential to enhance lifestyle management among people with multimorbidity.

## Introduction

Multimorbidity, the co-occurrence of two or more chronic conditions, has become recognized as a common medical condition and challenges health-care systems worldwide [1,2]. Multimorbidity is positively associated with increasing age [1, 3], high mortality [4, 5], functional decline [6, 7], and increased healthcare utilization [8, 9]. With the rapidly rising prevalence of multimorbidity, the paradigm of “one patient–one disease” no longer fits the medical necessities and needs of most patients. Patients with multimorbidity need a broader, more generic approach as the use of many services or treatment regimens to manage individual diseases can become duplicative, inefficient or even unsafe [1, 10, 11].

Due to limited evidence on available integrated and multidimensional care pathways for multimorbid patients, a care model for transitions in organizations and the delivery of care was recently developed [12]. The promotion of healthy lifestyle as a generic prevention strategy was one of the components in this model. Dietary behavior is among the most fundamentally important of health influences [13–15], but nutrition science devoted little study to diet and the coexistence of multiple chronic conditions in a single patient [16]. As a result, evidence on the impact of diet on a broad spectrum of chronic diseases is warranted to inform implementation strategies of dietary interventions in multimorbidity prevention and management.

Nutritional health outcomes are frequently the consequence of multiple synergies among nutrients and/or foods rather than just the (sum of) individual components [17]. Dietary pattern approaches recognizes complex combinations and interactions in the diet, an approach which paralleled by the transition from the era of “single chronic disease medicine” to the era of “multimorbidity medicine” [10, 11]. Focusing on isolated diseases or nutrients/foods cannot account for all interactions, and may result in erroneous conclusions [17, 18]. While for some affected disease domains strong associations with dietary patterns are thoroughly described [19, 20], an association with multimorbidity is yet to be established.

The objective of this study is therefore to examine the association between multimorbidity within the cardiometabolic disease domains and dietary patterns in the large representative adult population in the Netherlands.

## Subjects and methods

### Lifelines cohort study

All adult participants from the Lifelines cohort with reliable dietary intake were included in the present cross-sectional analysis (n = 129 369). Figure A in S1 File shows the flowchart diagram of participant selection. Lifelines is a population-based cohort study using a unique three-generation design to study the health and health-related behaviors of 167 729 persons living in the North of The Netherlands. The Lifelines population is broadly representative for the people living in this region [21]. Detailed information on the cohort profile can be found elsewhere [22]. In brief, individuals living in the recruitment area aged between 25 and 50 year, were invited through their general practitioners and inhabitants of the Northern provinces could register themselves via the Lifelines website. Participants received a baseline questionnaire and an invitation to a health assessment at one of the Lifelines research sites. During these visits, participants were asked whether their family members would also be willing to participate. Overall, 49% of the participants (n = 81 652) were invited through their GP, 38% (n = 64 489) via participating family members and 13% (n = 21 588) self-registered via the Lifelines website. Before study entry, all participants signed an informed consent. The Lifelines Cohort Study is conducted according to the principles of the Declaration of Helsinki and is approved by the medical ethics committee of the University Medical Center Groningen, The Netherlands.

### Multimorbidity score

Single morbidities within the cardiovascular, endocrinologic and renal domain were scored according to the 10th edition of the International Statistical Classification of Diseases and Related Health Problems (ICD-10) [23]. This method is in line with the study of Meems et. al [3], and a detailed description of the single morbidities within each disease domain can be found in Section A in S1 File. We calculated a simple morbidity score, the cardiometabolic morbidity score (CMMS), as a composite end-point, in which the cardiovascular, endocrinologic and/or renal domain is considered as ‘affected’ when at least one single disease is present within this disease domains shortly before and during the first visit at Lifelines outpatient clinic. Unfortunately, this method of registration has inherent uncertainties concerning sensitivity of disease registration. Therefore, self-reported diseases (or chronic conditions) were registered in this study when the use of appropriate medication was verified.

### Dietary assessment

To assess dietary intake in the Lifelines Cohort, a 110-item semi-quantitative baseline food frequency questionnaire (FFQ) assessing food intake over the previous month was developed by the Wageningen University using the Dutch FFQTOOL, in which food items were selected based on the Dutch National Food Consumption Survey of 1997/1998 [24]. The Lifelines FFQ was designed to include food groups that account for at least 80% of the variance and 80% of the population intake of both energy and macronutrients. Seven answer categories were used to assess consumption frequency, ranging from ‘not this month’ to ‘6–7 days a week’. Portion size was estimated by fixed portion sizes (e.g. slices of bread, pieces of fruit) and commonly used household measures (e.g. cups, spoons). Energy intake was estimated from the FFQ data by using the Dutch food composition database of 2011 [25]. Alcohol consumption was also estimated based on FFQ data. The reliability of reported dietary intake was based on the Goldberg cut-off method, which relies on the ratio of reported energy intake and basal metabolic rate [26], calculated with the Schofield equation [27]. A total of 14 726 participants with a ratio below 0.87 or above 2.75 were excluded (<0.89 or >2.66 for participants >75 years).

### Dietary pattern analysis

Previously, we identified four dietary patterns within this population. A detailed description of the methodology used to derive these patterns can be found elsewhere [28]. In short, dietary patterns were derived on the basis of principal components analysis (PCA) on the basis of 33 food groups (Table A in S1 File). The dietary patterns were derived on the basis of consumption (g/day) of each food group, unadjusted for energy intake. Within the PCA, orthogonal rotation (varimax option) was used to obtain uncorrelated patterns with greater interpretability. A component score was created for each of the dietary patterns identified by multiplying the factor loadings by the corresponding standardized intake of the food (standardized for men and women separately), and summing across the food items/groups for each pattern. Stability of the derived components was assessed by comparing the components solutions and factor loadings in two random halves of the data set and per sex group. Factor scores for each dietary pattern were categorized into quintiles (representing very low, low, moderate, high, and very high adherence to the dietary patterns).

### Sociodemographic, lifestyle characteristics and BMI

Self-administered questionnaires were used to collect data regarding demographics (education) and lifestyle (smoking, physical activity). Education was classified low education (primary school, vocational and lower general secondary education), moderate education (higher secondary education and intermediate vocational training), high education (higher vocational education and university education). Smoking status was categorized into non-smoker and current smoker. Physical activity was assessed using the short questionnaire to assess health-enhancing physical activity [29] and total moderate-to-vigorous physical activity was calculated in minutes per week. Height and body weight without shoes and heavy clothing were measured at one of the Lifelines research sites, with the SECA 222 stadiometer and the SECA 761 scale. Body mass index (BMI) in kg/m2 was calculated.

### Statistical analysis

Gender stratified baseline characteristics are presented according to categories of affected disease domains of the CMMS. Continuous data are represented as means ± standard deviation (SD) for normally distributed data and as medians with interquartile ranges (IQR) for non-normal distributions. Discrete and categorical data are presented as frequencies (%).

To study the association between quintiles of the dietary patterns scores and the CMMS we ran a generalized ordered logistic regression. This model relaxes the proportional odds assumption and allows the effects of the explanatory variables to vary with the point at which the categories of the dependent variable are dichotomized (partial proportional odds). In multivariate analysis, we created a model to adjust for risk factors for morbidity and determinants of diet. This model controlled for several determinants of both multimorbidity and diet (sex, age, smoking, physical activity, educational level, energy intake). In a second model body mass index (BMI) was additionally adjusted for. Odds ratios (ORs) were reported with 95% confidence intervals (95%CI). All *P* values are two-tailed. A *P* value < 0.05 was considered statistically significant. Analyses were performed using STATA v.11SE (College Station, TX, USA).

## Results

This study included 129 369 participants (41.5% males, 58.5% females) with a mean age of 44.8 (SD = 13.1, range 18–93). With increasing CMMS, subjects were more likely to be older, active smokers, lower educated, less active, to have a lower total energy intake and a higher prevalence of overweight and obesity (Table 1 and Table 2). The overall prevalence of cardiovascular, renal and endocrinologic diseases was 19.1%, 1.17% and 21.0% among males and 15.7%, 0.8% and 19.2% among females.

**Table 1:** Baseline data of according to the CMMS among males.

		CMMS			
morbidities		0	1	2	3
**N (%)**	** **	36451 (67.9)	12443 (23.2)	4569 (8.5)	189 (0.4)
**Age, year, mean (SD)**		42(12)	50(12)	59(11)	62(12)
**Age, %**	**<40**	41.7	16.3	3	4.2
** **	**40–60**	49.2	58.7	45.3	32.8
** **	**>60**	9.1	25.1	51.8	63
**Current smoker, %**	**yes**	23.9	21.3	17.0	25.4
**Education, %**	**Low**	25.4	34.5	44.2	46.5
** **	**Middle**	40.5	35.9	30.1	27.6
** **	**High**	34.1	29.6	25.8	25.9
**Weight status, %**	**BMI<25**	43.9	26.7	15.8	14.8
** **	**BMI 25–30**	45.8	54.8	55.8	47.6
** **	**BMI>30**	10.4	18.5	28.4	37.6
**Total moderate to vigorous physical activity (min/wk), mean (IQR)**		250 (60–600)	240 (50–540)	210 (30–480)	155 (0–445)
**CVD (%)**		NA	44.6	98.4	100
**CKD (%)**		NA	1.97	4.27	100
**Endocrine (%)**		NA	53.4	97.3	100

Low education = primary school, vocational and lower general secondary education. Moderate education = higher secondary education and intermediate vocational training. High education = higher vocational education and university education.

**Table 2:** Baseline data of according to the CMMS among females.

		CMMS			
morbidities		0	1	2	3
**N (%)**		54075 (71.4)	16846 (22.3)	4674 (6.2)	122 (0.2)
**Age, year (SD)**		57(12)	41(12)	51(12)	59(10)
**Age, %**	**<40**	43.1	16.3	2.9	6.6
	**40–60**	50.3	57.6	45	45.1
	**>60**	6.6	26.1	52.1	48.4
**Current smoker, %**	**yes**	20.4	17.3	14.8	21.3
**Education, %**	**Low**	23.7	39.8	59.6	62.2
	**Middle**	43.5	36.7	26.3	21.8
	**High**	32.8	23.5	14.1	16
**Weight status, %**	**BMI<25**	56.1	40.3	23.3	16.4
	**BMI 25–30**	31.2	38.2	41.7	36.1
	**BMI>30**	12.7	21.5	35	47.5
**Total moderate to vigorous physical activity (min/wk)**		180 (30–420)	225 (75–480)	210 (60–465)	200 (60–435)
**Total energy intake (kcal/d)**		1731 (482)	1858 (477)	1802 (464)	1727 (453)
**CVD (%)**		NA	39.6	99.0	100
**CKD (%)**		NA	2.0	2.6	100
**Endocrine (%)**		NA	58.4	98.3	100

Low education = primary school, vocational and lower general secondary education. Moderate education = higher secondary education and intermediate vocational training. High education = higher vocational education and university education.

### Dietary patterns

Detailed information on the dietary patterns that are derived from the data are described elsewhere (28). In short, four dietary patterns were retained in a PCA, together explaining 26.6% (7.6%, 7.0%,6.4% and 5.6% respectively) of the variation in food intake within this population (Table 3). The patterns were labels by the foods that correlated highly to the dietary patterns, i.e. a “bread and cookies” pattern, “snack” pattern, “meat and alcohol” pattern and a “vegetables, fish and fruit” pattern. The dietary patterns were stable and cross-classification of quintiles of gender specific standardized dietary pattern scores to non-gender specific standardized dietary pattern scores showed 97% of participants was categorized in the same or adjacent quintile (results not shown).

**Table 3:** Factor loading * matrix of the four identified dietary patterns in the entire study population.

Bread and sweets		Snacks		Meat, alcohol and potato		Vegetable, fish and fruit	
Food groups	Factor loading	Food groups	Factor loading	Food groups	Factor loading	Food groups	Factor loading
Low-fat margarine /margarine/butter	0.69	Other snacks	0.67	Processed meat	0.56	Vegetables	0.57
Bread and bread products	0.68	Pizza	0.54	Fresh meat	0.56	Fish and seafood	0.49
Sugar and confectionary	0.58	Ready to serve dinner	0.49	Alcoholic drinks	0.49	Rice/pasta	0.47
Potatoes	0.54	French fries	0.49	Coffee	0.47	Legumes	0.45
Cake and cookies	0.44	Sugar sweetened beverages	0.47	Chicken	0.43	Fruit	0.41
Sauces/dressing/gravy	0.43	Fruit/vegetable juices	0.30	Sauces/dressing/gravy	0.34	Nuts and seeds	0.35
High fat dairy products	0.36	Rice/pasta	0.27	Eggs	0.27	Eggs	0.30
Processed meat	0.31	Sauces/dressing/ggravy	0.26	Potatoes	0.25	Breakfast cereals	0.30
Sugar sweetened beverages	0.22	Savory bread topping	0.26	Fruit	-0.21	Soup	0.26
Fish and seafood	-0.26	Sugar and confectionary	0.24	Cake and cookies	-0.22	Tea	0.25
		Alcoholic drinks	0.21	Tea	-0.48	Sugar sweetened beverages	-0.22
		Vegetables	-0.24				
		Fruit	-0.33				

* factor loadings > 0.2

### Association between quintiles of dietary pattern with CMMS

**Fig 1:**
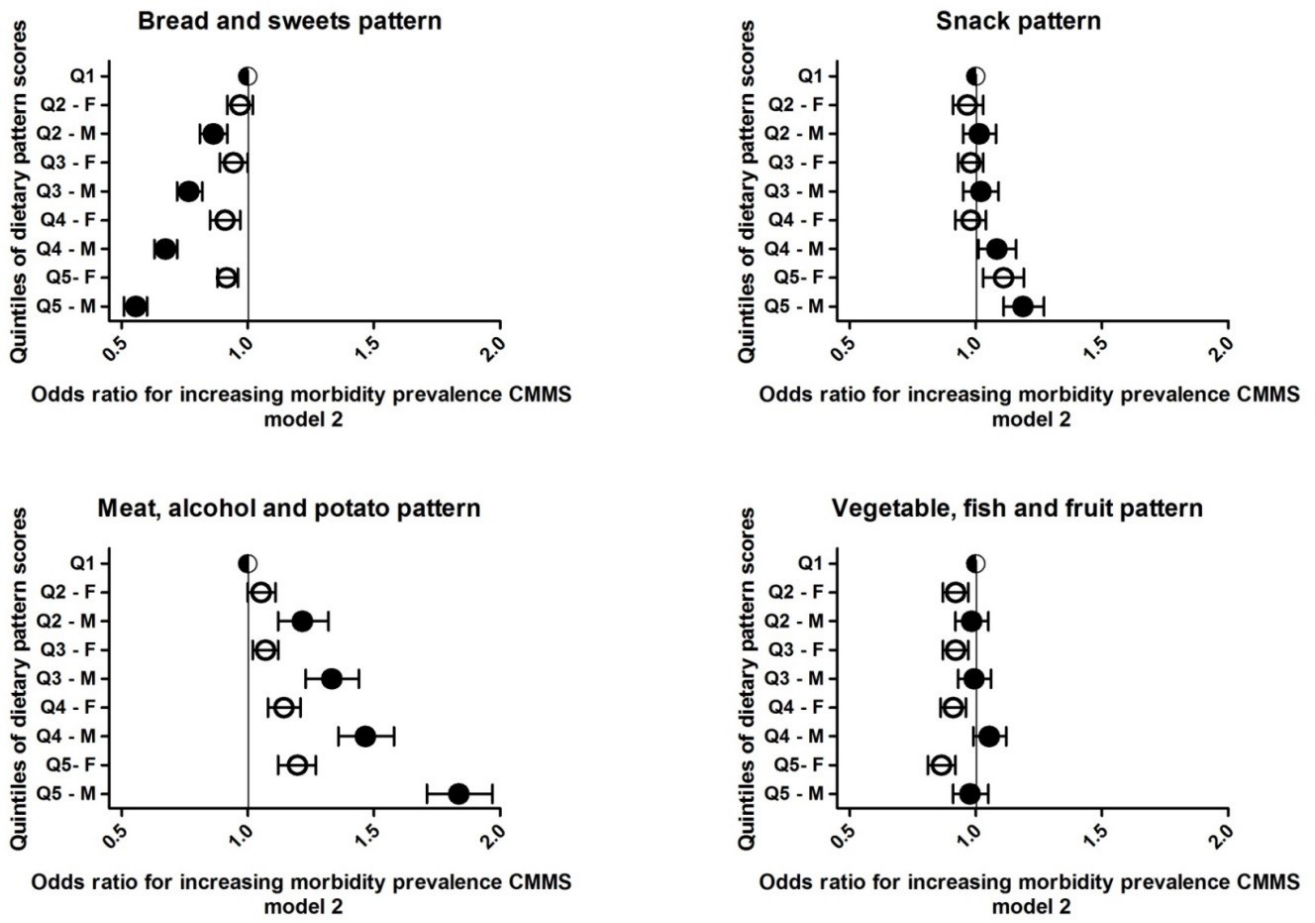
The association between quintiles of four dietary patterns scores and CMMS odds ratio with 95% CI. Black dots = male, white dots = female; Model 2: age, sex, physical activity, smoking, educational attainment, energy intake.

Significant differences in the association between quintiles of all four dietary pattern scores and the CMMS according to gender were observed (P for interaction ≤0.001, results not shown). Therefore, the results are presented for men and women separately (Table 4 and Table 5). Results from model 2, presented in Fig 1, showed a significant inverse association between the quintiles of the “bread and sweets pattern” and the CMMS. The opposite was observed with respect to the “meat, alcohol and potato pattern” and the “snack pattern” in which greater adherence was associated with a higher odds of having a CMMS, especially among men. To illustrate, if a man with a high consumption of foods typical for a “meat, alcohol and potato pattern” changed his diet to a pattern that was not characterized by these foods he would reduce his chance for a higher morbidity score with 83%. While higher adherence to the “vegetable, fish and fruit pattern” was associated with a lower odds of multimorbidity, after adjustment for BMI the significant trend disappeared.

**Table 4:** Multivariate association of dietary patterns with CMMS among males.

Models *		Model 1	Model 2	Model 3
		OR (95%CI)	OR (95%CI)	OR (95%CI)
**Bread and sweets (Q1 = ref)**	2	0.87 (0.82–0.93)	0.86 (0.81–0.92)	0.87 (0.82–0.93)
	3	0.78 (0.73–0.84)	0.76 (0.72–0.82)	0.80 (0.74–0.85)
	4	0.69 (065–0.74)	0.67 (0.63–0.72)	0.73 (0.68–0.78)
	5	0.58 (0.54–0.62)	0.56 (0.51–0.60)	0.65 (0.60–0.70)
**p for trend**		**≤0.001**	**≤0.001**	**≤0.001**
**Snack (Q1 = ref)**	2	1.00 (0.94–1.06)	1.01 (0.95–1.08)	1.00 (0.94–1.06)
	3	0.98 (0.92–1.05)	1.02 (0.95–1.09)	0.99 (0.93–1.06)
	4	1.01 (0.95–1.08)	1.08 (1.01–1.16)	1.05 (0.98–1.13)
	5	1.04 (0.97–1.11)	1.18 (1.11–1.27)	1.12 (1.04–1.20)
**p for trend**		0.213	**≤0.001**	**≤0.001**
**Meat, alcohol and potato (Q1 = ref)**	2	1.25 (1.15–1.35)	1.21 (1.12–1.32)	1.12 (1.03–1.22)
	3	1.37 (1.27–1.48)	1.33 (1.23–1.44)	1.20 (1.11–1.30)
	4	1.50 (1.40–1.62)	1.46 (1.36–1.58)	1.26 (1.17–1.36)
	5	1.81 (1.69–1.95)	1.83 (1.71–1.97)	1.48 (1.37–1.59)
**p for trend**		**≤0.001**	**≤0.001**	**≤0.001**
**Vegetable, fish and fruit (Q1 = ref)**	2	0.92 (0.86–0.98)	0.98 (0.92–1.05)	0.98 (0.92–1.05)
	3	0.88 (0.83–0.94)	0.99 (0.92–1.06)	0.99 (0.93–1.06)
	4	0.89 (0.84–0.95)	1.05 (0.99–1.12)	1.08 (1.00–1.15)
	5	0.76 (0.72–0.81)	0.97 (0.91–1.05)	1.00 (0.94–1.08)
**p for trend**		**≤0.001**	0.818	0.165

* Model 1 includes age, model 2 includes model 1+physical activity, smoking, energy intake and educational attainment, model 3 includes model 2 + BMI.

**Table 5:** Multivariate association of dietary patterns with CMMS among females.

Models *		Model 1	Model 2	Model 3
		OR (95%CI)	OR (95%CI)	OR (95%CI)
**Bread and sweets (Q1 = ref)**	2	0.97 (0.92–1.01)	0.97 (0.92–1.02)	0.98 (0.93–1.04)
	3	0.93 (0.88–0.97)	0.94 (0.89–1.00)	0.97 (0.92–1.02)
	4	0.88 (0.84–0.93)	0.91 (0.85–0.97)	0.96 (0.90–1.03)
	5	0.81 (0.76–0.86)	0.88 (0.81–0.96)	0.94 (0.86–1.02)
**p for trend**		**≤0.001**	**≤0.001**	0.176
**Snack (Q1 = ref)**	2	0.94 (0.89–0.98)	0.96 (0.91–1.01)	0.97 (0.93–1.02)
	3	0.94 (0.89–0.99)	0.98 (0.93–1.03)	0.98 (0.93–1.04)
	4	0.92 (0.87–0.98)	0.98 (0.93 (1.04)	0.98 (0.92–1.04)
	5	1.00 (0.94–1.07)	1.11 (1.03 (1.19)	1.06 (0.98–1.13)
**p for trend**		**≤0.001**	**0.04**	0.349
**Meat, alcohol and potato (Q1 = ref)**	2	1.07 (1.02–1.13)	1.05 (1.00–1.11)	1.00 (0.95–1.05)
	3	1.10 (1.05–1.16)	1.07 (1.02–1.12)	0.98 (0.93–1.03)
	4	1.18 (1.12–1.24)	1.14 (1.08–1.21)	1.01 (0.96–1.07)
	5	1.23 (1.16–1.31)	1.20 (1.12–1.27)	1.01 (0.95–1.08)
**p for trend**		**≤0.001**	**≤0.001**	0.53
**Vegetable, fish and fruit (Q1 = ref)**	2	0.87 (0.83–0.93)	0.92 (0.87–0.97)	0.94 (0.89–1.00)
	3	0.84 (0.79–0.88)	0.92 (0.87–0.97)	0.95 (0.90–1.01)
	4	0.81 (0.76–0.85)	0.91 (0.86–0.96)	0.96 (0.91–1.02)
	5	0.72 (0.68–0.76)	0.86 (0.81–0.92)	0.94 (0.88–0.99)
**p for trend**		**≤0.001**	**≤0.001**	0.148

* Model 1 includes age, model 2 includes model 1+physical activity, smoking, energy intake and educational attainment, model 3 includes model 2 + BMI

## Discussion

We demonstrate in this cross-sectional study that empirically derived dietary patterns, after adjustment for potential confounders, are associated with the prevalence of multimorbidity within the cardiometabolic disease domain among men and women. In general, higher adherence to a “meat, alcohol and potato pattern” and a “snack pattern” was associated with a higher odds of having more affected disease domains within the individual, while adherence to a “bread and sweets pattern” and to a lesser extent a “vegetable, fish and fruit pattern” was inversely associated with the CMMS. The strength of the associations largely depended on gender. After adjustment for BMI, the associations were attenuated, but remained significant for most dietary patterns among men.

Targeting specific dietary patterns might favor dietary change as modification will be more readily achieved if recommended foods are compatible with existing patterns of food consumption [30–32]. This study is the first to show the associations between empirically derived dietary patterns and prevalence of multimorbidity. The results of this study suggest that higher adherence to a dietary pattern characterized by high intakes of meat, alcohol and potato and also a “snack pattern” is associated with increased prevalence of multimorbidity within the cardiometabolic domain. While previous work has substantially contributed to the understanding and importance of the study of dietary patterns with respect to single outcomes, disentangling the potential influence of dietary patterns on health from factors such as the totality of someone’s disease profile is challenging, if not impossible. To illustrate, Rodriguez-Monforte et. al and Li et. al found the rather unexpected finding that no pooled association between the adherence to unhealthy dietary patterns and stroke mortality was present [19, 33]. This could be explained by the fact there was large heterogeneity in foods that characterized this “unhealthy pattern”, but also that the adherence to such pattern could be associated with a higher risk of other diseases such as cancer, myocardial infarction and ischaemic heart disease that might lead to death before a stroke could occur.

We found that a diet characterized by high intakes of vegetables, fish and fruit was associated with a modest decrease in the likelihood of higher CMMS. This finding is in line with a previous study in which greater consumption of foods such as fruits and vegetable and whole grain products appeared to lower the risk of multimorbidity [16]. The association, however, disappeared after adjustment for BMI. Several explanations are proposed, e.g. higher BMI is associated with selective under-reporting of certain foods (such as unhealthy snacks), and over-reporting of presumably more “healthy” foods [34]. Additionally, as obesity may be an intermediate step in the pathway between diet and some affected diseases domains, one can argue that the models adjusted for BMI present an overadjustment. Obesity is associated with increased frequency of many long-term conditions that are of importance in primary and secondary care, including type 2 diabetes, cardiovascular diseases and musculoskeletal problems [35, 36]. Several studies have reported that obesity is a risk factor for multimorbidity in middle-aged and older adults. For example, Agborsangaya et al found that having obesity was associated with more than double the odds of multimorbidity (odds ratio = 2.2, 95% CI 1.9–2.7) compared to non-obese [37]. Using population attributable fractions, it has been estimated that almost a third of multimorbidity could be attributable to overweight and obesity, and a fifth to obesity alone; a causal relationship can be assumed [35]. BMI assessment to identify and monitor obesity should be a priority for those working in health care services, along with weight and lifestyle management and targeted control of other risk factors such as hypertension and hypercholesterolaemia. Such changes in practice could potentially reduce the onset and burden of multimorbidity.

In our study higher adherence to a “bread and sweets pattern” was associated with lower multimorbidity scores, especially in men. This finding is somewhat counterintuitive, but it is in line with the observation that higher intakes of a dietary pattern characterized by high intakes of cakes and cookies and whole-grain bread was found to be significantly associated with a lower risk of chronic diseases [38]. Additionally, cake and cookies were found to be positively associated with cardiorespiratory fitness [39]. This observation may have a biological background, or could be the result of some type of reporting bias, in which people with higher multimorbidity scores tend to under-report their bread and sweets intake compared with their counterparts. Another well-established source of bias, namely “confounding by indication” might also apply, as cross-sectional data are prone to this type of bias. It implicates that individuals at risk have already adapted their dietary habits based on medical or preventive advice. At this point, we can only conclude that the observation is interesting and that this issue should be investigated further, preferably in longitudinal studies.

Gender-specific differences were observed. In general, the associations were stronger for men than women, except for the vegetable, fish and fruit pattern. Hypothetically, this may be explained by the fact that men and women show heterogeneity in diet intake [13, 40]. In our population, we found no reason to assess dietary patterns separately for men and women as in stratified analysis the same patterns were observed. We did however, calculate dietary pattern scores on the basis of gender specific standardized intakes of foods to minimize the effect of lower total intake in women. Still, we observed striking gender differences in the adherence to dietary patterns, being most outspoken for the “meat, alcohol and potato pattern”. Only a small proportion of women was assigned to the highest adherence group (10%), against a relatively large proportion of men (34%). Women and men may have completed the FFQ differently, resulting in different degrees of measurement error which may affected the assessment of food intake and consequently the definition of dietary patterns [41, 42]. Still, it is warranted to acknowledge gender differences in dietary patterns in relation to disease outcomes to optimally inform future dietary recommendations. It might well be that recommending to eat more of the healthy foods for women, and less of the unhealthy foods for men would be an approach to stimulate effective dietary change and consequently decrease disease risk [43].

The present study has several limitations. First, although diet was assessed through a validated FFQ, the dietary analysis relied on self-reported data which may affected the patterns found through PCA. Additionally, PCA requires several arbitrary decisions [44]. Yet, it was found that derived dietary patterns were robust for subjective factor analytical decisions. With respect to diet, we excluded participants with implausible energy intake. Second, although clinical examination was included, some of the diseases or conditions were self-reported, and this may have caused under- or over-reporting of these morbidities. However, in the current framework of the Lifelines Cohort Study we considered this approach most feasible, although we acknowledge limitations inherent to this approach. Another limitation of this study may be the simplified composite end-point of morbidity score. Although we recognize that the effect of multimorbidity on individuals will vary with the combination and severity of morbidities, we chose to weight all morbidities equally, which resulted in a simplified count to define morbidity. This approach is different from other scores, like the Charlons co-morbidity score [45] that also takes into account the severity of the co-morbidity. Of note, we are not the first ones to use such an unweighted score [3, 46], this strategy enabled us to include morbidities that have a low prevalence. Another limitation is that this is a cross-sectional study, hereby these analyses do not provide evidence about possible causality of the adherence to dietary patterns and multimorbidity prevalence. As suggested by XU et.al, there is a need for prospective research, especially longitudinal cohort studies and randomized control trials, to provide more definitive evidence on multimorbidity [47]. Last, this study has an observational nature. Despite the fact that we adjusted for potential confounders, we cannot exclude a possible effect of any unmeasured factors on the observed association. This study has several strengths such as the availability of detailed data on diet and health among a very large homogeneous representative population of men and women. We used standardized definitions of specific morbidities when possible (i.e. ICD-10), explicitly reported elsewhere [3].

To conclude, robust associations between dietary patterns and multimorbidity, in particular a “meat, alcohol and potato pattern” within the cardiometabolic domain, suggest an important opportunity of dietary interventions in multimorbidity prevention and management. These results therefore provide an opportunity for the implementation of dietary pattern interventions as a generic lifestyle strategy within the care of patients with multimorbidity.

## Supporting information

S1 File (Figure A) Flowchart diagram of participant selection. (Section A) Detailed description of the single morbidities within each disease domain. (Table A) Foods and food groups used in the dietary pattern analysis.
(DOCX)
