# Supplementary material for: The association of multimorbidity within cardio-metabolic disease domains with dietary patterns: A cross-sectional study in 129 369 men and women from the Lifelines cohort
Source: PLoS One. 2019 Aug 8;14(8):e0220368. doi: 10.1371/journal.pone.0220368 (PMC6687151; doi:10.1371/journal.pone.0220368)
Supplement: S1 File — (Figure A) Flowchart diagram of participant selection. (Section A) Detailed description of the single morbidities within each disease domain. (Table A) Foods and food groups used in the dietary pattern analysis. (DOCX) [file pone.0220368.s001.docx]

**S1 File**

**Figure A.** **Flowchart diagram of participant selection**

Excluded

N=14 726 without reliable FFQ data

Excluded

N=8 587 participants without FFQ data

Excluded

N=15 067 children (≤ 17 years

Total Lifelines population

N=167 729

Adults (≥ 18 years)

N=152 662

Adults with FFQ data

N=144 095

Reliable FFQ data*

N=129 369

* The reliability of reported dietary intake was based on the Goldberg cut-off method.

**Section A. Detailed description of the single morbidities within each disease domain**

**ENDOCRINOLOGIC DISEASE**

Diabetes (IV-E10/E11/E12/E14)

- Self-reported diabetes or

- use of oral anti-diabetics and/or insulin or

- Fasting glucose levels >6.99 or

- Non-fasting glucose level >11.0

NB: we defined different types of diabetes and excludes diabetes related to pregnancy, but not to any (other) medical cause.

Thyroid disease (IV-E03/E05)

- Use of thyroid hormones or

- 6-TSH>4 & FT4<11.0 (hypothyroidism) or

- TSH>10 or -TSH <0.5 & FT4>19.5 (hyperthyroidism) Hypercholesterolemia (IV-E78.0)

- Self-reported myocardial infarction and cholesterol >=5.0 or

- Use of lipid lowering medication or

- Cholesterol >= 6.5 mmol/L

Oral anti-diabetics= ATC-code A10B insulin= ATC-code A10A thyroid hormones= ATC-code H03 lipid lowering drugs= ATC-code C10A, C10B

Morbidity within the endocrinologic disease domain is present if one of the following diseases occurs:

- diabetes

- thyroid disease

- hypercholesterolemia

**CARDIOVASCULAR DISEASE**

Hypertension (IX-I10) is present, if

- BP> 140/90mmHG or

- use of anti-hypertensive medication

Heart failure (IX-I50) is present, if

- self-reported disease, and

- use of (any) HF-related medication (diuretics, ACE-I, aldosteronantagonist, betablocker, angiotensin receptor blocker)

Vascular disease (IX-I21, IX-I25.2, IX-I64) is present, if

- Self-reported MI, stroke, PCI & CABG, and

- use of vitamin K antagonists or ascal/acetylsalicylic acid or clopidogrel

- control: and use of (simva-)statin (however, 10% of this patient group does NOT use (simva-)statin)

Atrial fibrillation (IX-I48) is present, if

- self reported, ‘diagnosed by MD’ and use of vitamin K antagonists, or

- AF on ECG and use of vitamin K antagonists, or

- CHADVASC <2 and AF on ECG

anti-hypertensive medication= ATC-code C02, C03, C04, C07, C08, C09 diuretics= ATC-code C03A, C03B, C03C, C03E, C03X aldosteronantagonist= ATC-code C03D betablocker= ATC-code C07 calcium antagonist= ATC-code C08 ACE-inhibitors and ANG II antagonists = ATC-code C09 Vitamin K antagonists = ATC-code B01AA Ascal = ATC-code B01AC06 Acetylsalicylic acid= ATC-code B01AC08 clopidogrel= ATC-code B01AC04 statin= ATC-code C10AA simvastatin= ATC-code C10AA01

Cardiovascular disease is present, if one of the following disease symptoms occurs:

- Heart failure

- Atrial fibrillation

- Hypertension

- Vascular disease

**RENAL DISEASE**

Impaired renal function (XIV-N19) is present, if

- eGFR ≥ 60 ml/min/1.73m2 with albuminuria (24h albumin >30)

- eGFR <60 ml/min/1.73m2

Morbidity within the renal disease domain is present, if one of the following disease symptoms occurs:

Impaired renal function

**Table A. Foods and food groups used in the dietary pattern analysis**

| Food groups | Foods |
| --- | --- |
| Alcoholic drinks | Beer, wine and fortified wine, white-, rosé and red wine, sherry, port wine or vermouth, spirits, distilled drinks (jenever, whisky, rum, gin, cognac, vieux, liqueur) |
| Bread and bread products | Bread, crispbread, rusk, croissants and others |
| Breakfast drink | Breakfast drink |
| Butter, margarine, low fat margarine | Butter, margarine, low fat margarine |
| Cakes and cookies | Small cookies or (nutritional) biscuits, sponge cake, large cookies, cake, pie |
| Cereals | Muesli, granola or cereals for the preparation of porridges |
| Chicken | Chicken with and without skin |
| Coffee | Coffee |
| Commercially prepared dishes | Chinese/Indonesian dishes, meals from fast-food restaurants, other types of ready-to-eat meals |
| Diet beverages | Diet soft drinks or lemonade without sugar |
| Eggs | Boiled and fried eggs |
| Fish and seafood | Salted herring, fried herring, salmon, mackerel, eel, cod, plaice, haddock, pollack, sole, deep-fried whiting in dough etc. |
| Fries | Fried potatoes |
| Fruit | Fruit and apple sauce |
| Fruit and fruit/vegetable juices | Fruit and vegetable juices |
| Savory bread toppings | Savory bread toppings |
| High fat milk products | 40+ or 48+ cheese or spreadable cheese, cream cheese and/or foreign cheese, (full-fat) custard and other milk-based desserts, milk-based ice cream, Butter milk, full-fat plain yogurt, semi-skimmed plain yogurt, skimmed plain yogurt, chocolate milk, quark or fruit quark |
| Legumes | Brown beans, white beans, marrowfat peas, kidney beans etc. |
| Low fat milk products | 20+ or 30+ cheese or spreadable cheese, nonfermented medium/ low-fat milk or yoghurt |
| Non-alcoholic drinks | Alcohol-free beer |
| Nuts and seeds | Peanuts, nuts and seeds |
| Other snacks | Croquettes, minced meat hot dogs, sausage rolls, Savory snacks, potato chips or salty biscuits |
| Pizza | Pizza |
| Potatoes | Boiled or mashed potatoes |
| Processed meat | Luncheon meats, hamburger, minced meat (beef or mix of beef and pork), smoked sausages or frankfurters |
| Red meat | Sirloin steak, beef bratwurst, beef blade steak, beef rib steak or marbled beef, bacon, pork bratwurst, ‘slavink’ (ground meat wrapped in bacon), pork, beef steak, steak tartare, braising steak or roast beef |
| Rice /pasta | Pasta, rice |
| Sauces | Pasta sauce, mushroom sauce, sate sauce |
| Sauces /dressing/gravy | Regular mayonnaise, low fat mayonnaise, sauce for French fries and other non-red sauces, gravy, salad dressing with/without oil |
| Soup | Soups (with and without legumes) |
| Sugar and confectionery | Chocolate, candy with chocolate and chocolates, liquorice, acid drops etc., sweet sandwich toppings (chocolate- sprinkles, spread or flakes, honey, jam), sugar, honey or syrups, candy bars (Mars, Snickers, M&M's etc.) |
| Sugar sweetened beverages | Sugar sweetened beverages such as soft drinks (coke, orange flavored soft drinks, 7-up) or lemonade with sugar or sugar sweetened milk/yoghurt drinks |
| Tea | Tea |
| Vegetables | Vegetables |
